# Tissue-Resident Macrophages Limit Pulmonary CD8 Resident Memory T Cell Establishment

**DOI:** 10.3389/fimmu.2019.02332

**Published:** 2019-10-10

**Authors:** Nick P. Goplen, Su Huang, Bibo Zhu, In Su Cheon, Young Min Son, Zheng Wang, Chaofan Li, Qigang Dai, Li Jiang, Jie Sun

**Affiliations:** ^1^Thoracic Diseases Research Unit, Division of Pulmonary and Critical Care Medicine, Department of Medicine, Mayo Clinic College of Medicine and Science, Rochester, MN, United States; ^2^Department of Immunology, Mayo Clinic College of Medicine and Science, Rochester, MN, United States

**Keywords:** tissue-resident memory, alveolar macrophage, CD8 T cell differentiation, influenza, PPAR-γ, CD69, CD169

## Abstract

Tissue resident memory CD8 T cells (T_RM_) serve as potent local sentinels and contribute significantly to protective immunity against intracellular mucosal pathogens. While the molecular and transcriptional underpinnings of T_RM_ differentiation are emerging, how T_RM_ establishment is regulated by other leukocytes *in vivo* is largely unclear. Here, we observed that expression of PPAR-γ in the myeloid compartment was a negative regulator of CD8 T_RM_ establishment following influenza virus infection. Interestingly, myeloid deficiency of PPAR-γ resulted in selective impairment of the tissue-resident alveolar macrophage (AM) compartment during primary influenza infection, suggesting that AM are likely negative regulators of CD8 T_RM_ differentiation. Indeed, influenza-specific CD8 T_RM_ cell numbers were increased following early, but not late ablation of AM using the CD169-DTR model. Importantly, these findings were specific to the parenchyma of infected tissue as circulating memory T cell frequencies in lung and T_CM_ and T_EM_ in spleen were largely unaltered following macrophage ablation. Further, the magnitude of the effector response could not explain these observations. These data indicate local regulation of pulmonary T_RM_ differentiation is alveolar macrophage dependent. These, findings could aid in vaccine design aimed at increasing T_RM_ density to enhance protective immunity, or deflating their numbers in conditions where they cause overt or veiled chronic pathologies.

## Introduction

Residual CD8 T cells from primary responses form a long-lived immunological memory barrier poised at multiple anatomical sites (1–5). CD8 resident memory T cells (T_RM_) precluded from the circulatory system migrating within either non-lymphoid or secondary draining lymphoid organs, represent nearly one-third of the total CD8 memory T cell pool following primary responses (6–9). Compared to circulating conventional memory T cells [i.e., effector memory (T_EM_) and central memory (T_CM_)], T_RM_ offer rapid local protection from pathogens (10, 11). This protective immuno-surveillance is highly dependent on T_RM_ density which can be greatly enhanced through *in situ* self-renewal, replenishment from circulating memory T cells, and *de novo* T cell differentiation following a secondary exposure (6–9, 12). Yet, little is known about the local cellular immune-networks that locally mediate differentiation and thereby regulate initial T_RM_ density in the lung and elsewhere.

CD8 T_RM_ begin their differentiation in secondary lymphoid organs in the context of TCR, co-stimulatory, and cytokine receptor signaling derived from sufficiently activated dendritic cells (13–17). Exogenous uptake of viruses or infected cells by DCs followed by cross-presentation of viral peptide to CD8 T cells in secondary lymphoid organs markedly enhances T_RM_ differentiation (18–23). Following priming, T_RM_ cells derive from the memory-precursor effector cell (MPEC) pool (17, 24). These early memory precursors (CD127^+^KLRG-1^Lo^, including ex-KLRG-1 MPECs) are not just precursors to T_RM_, but also T_CM_ (17, 24–27).

Remarkably, circulating memory CD8 T cells receive all the required cues provided by professional antigen presenting cells for appreciable clonal expansion and full functional differentiation *in vivo* within the first 3 days following an acute inflammatory infection (14, 17, 28–31). In contrast, T_RM_ commitment windows occur within 7–14 days and appear to be influenced by much later factors in the context of an inflamed tissue environment commensurate with exposure to TGF-β (27, 32–35). Additional TCR and CD28 signaling and cytokines such as IL-7, IL-15, IL-12, IL-18, IL-21, Type I interferons, and TNFa as well as interactions with stroma and extracellular matrix may be further epitope, tissue, or pathogen-specific requirements for T_RM_ differentiation and or maintenance (24, 36–46). Hence, CD8 T_RM_ undergo a second stage of differentiation at the site of infection and though context-dependent, exhibit distinct differentiation and maintenance requirements relative to their circulatory memory counterparts programmed early after activation (14, 24, 32, 46).

The cellular networks involved in this extra stage of differentiation from naive to MPEC CD8 T cell, to that which establishes the transcriptional program required for T_RM_ residency (43), are just now being worked out and the focus of this study. In a model of intestinal Yersinia *pseudotuberculosis* infection, inflammatory macrophages derived from bone-marrow monocytes (CCR2-dependent migration) accumulate and positively regulate the differentiation of CD103^−^ T_RM_ at the site of inflammation via provision of signal 3 cytokines (IL-12 and type I IFNs) that dampen CD103 expression (40, 47). Therefore, inflammatory cytokines provided by bone marrow-derived macrophages can endow heterogenous T_RM_ sentinel programming in the gut. Similarly, in vaccinia virus infection, inflammatory monocytes (Ly6c^hi^, CCR2-dependent) were responsible for long-term maintenance of a subset of pulmonary T_RM_ without affecting clonal expansion or contraction (48). Further, a network involving CD4 T_RM_ in the female reproductive tract (FRT) forms positive-feedback loops with local macrophages that promote low-level constitutive IFNγ production from the CD4 T_RM_ resulting in organization of local macrophage-mediated immune-clusters outside of secondary lymphoid tissue (49). Once residency is established, function of CD8 T_RM_ in the FRT depend on CD301b^+^ cDC2 to provide TCR signals in the lamina propria (50). Thus, a limited number of studies have implicated macrophages as positive regulators of CD4 and CD8 T_RM_ establishment, but the origins of the tissue-resident macrophages in question are not clear in all cases.

In this study, we investigated a genetic model in which the alveolar macrophage compartment has a known functional deficit in influenza responses and found this was associated with increased local CD8 memory T cell density. A model in which alveolar macrophages were ablated prior to infection, but not during T cell contraction, also exhibited enhanced flu-specific resident memory T cell density compared to controls. In contrast, no major alterations in the circulating T_EM_ or T_CM_ influenza responses were observed following alveolar macrophage ablation. This work suggests that manipulation of the alveolar macrophage compartment may be an attractive target for modulating T_RM_ density for therapeutics or vaccine design.

## Materials and Methods

### Mice and Infection

Lyz2-cre, and PPAR-γ^fl/fl^ *(C57BL/6) were* purchased from the Jackson (Harbor, ME) Laboratory and bred in house. C57BL/6 CD169-DTR (51, 52) were received from Professor Tanaka (Tokyo, Japan). In all cases, wild-type control mice were transgene or Cre-negative littermates. All mice were housed in a specific pathogen-free environment and used under conditions fully reviewed and approved by the internal animal care and use committee (IACUC, approval #A00002035) guidelines at the Mayo Clinic (Rochester, MN). For influenza virus infection, influenza A/PR8/1934 strain (~120 pfu/mouse) was diluted in FBS-free DMEM media (Corning) on ice and inoculated in anesthetized mice through intranasal route (35 μl) as described before (53). This low dose was chosen because higher, easily tolerable doses in wt animals leads to death in macrophage-depleted animals (54). Where applicable, mice were re-challenged on day 53 with influenza A/X-31 strain (1.2 × 10^5^ pfu/mouse) with daily treatment of FTY720 (25 μg/mouse) starting 1 day before re-challenge.

### Cell Depletions

For depletion of CD169-positive cells at time-points indicated in text and figure legends, CD169-DTR mice and DTR-littermate controls were injected with diphtheria toxin (Sigma, DTx, 300 ng/mouse) every 3 days from the time indicated. Data were analyzed at day 3, 10, or 22 post-infection (DTx treatment started day minus 1 or day 10 post-infection) to assess CTL response or depletion efficiency, or at day 42 or 53 to analyze effects of macrophage depletion on cellular makeup of the lung environment following intravital labeling of white blood cells in the circulation as described below.

### Tissue Processing, Cellular Isolation, and Data Analysis

Animals were injected intravenously with 3 μg of CD45 or CD8β antibody labeled with various fluorochromes. Two minutes post-injection, animals were euthanized with an overdose of ketamine/xylazine. Following euthanasia, spleens were removed and BAL samples were taken by repeated gentle instillation and removal of PBS via the trachea as previously reported (55). The right ventrical was gently perfused with PBS (10 mL). Lungs were instilled with 1 mL of digestion buffer [90%DMEM 10% PBS + Calcium and Magnesium with 180 U/mL Type 2 Collagenase (Worthington) and 15 μg/mL DNase (Sigma) additives]. Tissue was processed on a gentleMACS tissue disrupter (Miltenyi) for 40 min at 37°C followed by hypotonic lysis of red blood cells in ammonium-chloride-potassium buffer and filtering through 70 μm mesh. Fc-gamma receptors were blocked with anti CD16/32 (2.4G2). Cell surfaces were immuno-stained with following cocktails of fluorochrome-conjugated Abs (Biolegend) Siglec-F (E50-2440), CD11c (N418), CD11b (M1/70), merTK (2B10c42), CD64 (X54-4/7.1), Ly6G (1A8), I-A/I-E (M5/114.15.2), Ly6C (HK1.4), immuno-staining was performed at 4°C for 30 min. Cells were washed twice with FACS buffer (PBS, 2 mM EDTA, 2% FBS, 0.09% Sodium Azide), prior to fixation and ran on an Attune NxT auto sampler (Life Technologies). FCS files for myeloid stains were analyzed with FlowJo 10.2 (Tree Star) and processed in a similar way to previous reports (56) as shown in Supplementary Figure 1. Following intra vital labeling, single cell lung or spleen suspensions were immuno-stained with antibodies against: CD8α (53-6.7) CD69 (H1.2F3), CD44 (IM7), CD11a (M17/4), CD103 (2E7), PD-1 (29F.1A12), and NP_366−374_-D^b^ and PA_224−233_-D^b^ tetramers (NIH Tetramer Core Facility) on ice for 45–60 min. Following exclusion of doublets and gating on total CD8 T cells, residency was determined by increased CD69 expression on CD8α^+^ cells protected from circulatory labeling per Supplementary Figure 1. Nearly 100% of cells in the lung defined this way express hi levels of CD44 at the time-points investigated. CD103 was ignored in analyses because in the lung 6 weeks after infection, CD103 expression is T_RM_ epitope-specific (46). Central and Effector memory CD8 T cells (T_CM_ and T_EM_, respectively) were differentiated by CD62L expression (T_CM_ = Hi, T_EM_ = Lo) on splenic CD8 T cells at least 42 days post-infection that were D^b^-NP or -PA-tetramer^+^ CD44^Hi^ CD127^+^.

### Statistical Analysis

Quantitative data are presented as mean ± Standard of Deviation. Unpaired two-tailed Student's *t*-test (two-tailed, unequal variance) were used to determine statistical significance with Prism software (Graphpad). We considered α < 0.05 as significant in all statistical tests and denoted within figures as a ^*^.

## Results

### Myeloid PPAR-γ Deficiency Increases CD8 T_RM_ Establishment

**Figure 1:**
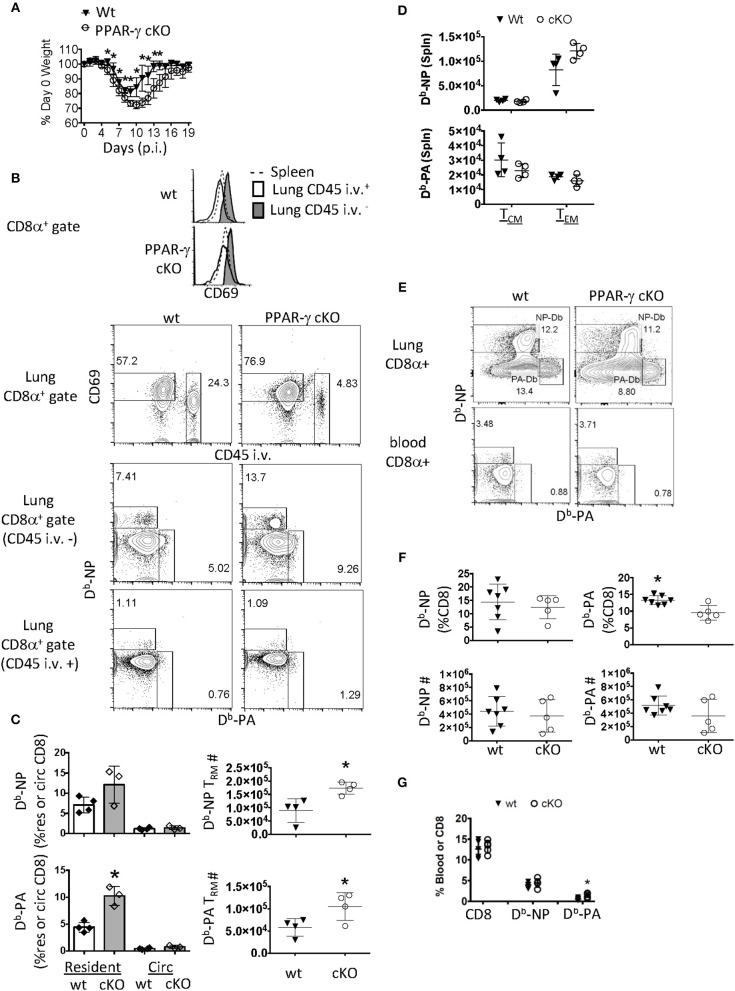
PPAR-γ (Lyz2-Cre cKO) mice exhibit enhanced flu-specific T_RM_ response. Wt C57BL/6 and PPAR-γ^fl/fl^ x Lyz2-Cre mice were infected with PR8 influenza virus. **(A)** Percentage of initial body weight was monitored from day 0 to 19. **(B)** CD8 T cells were assayed by flow cytometry day 60 post-infection. From lung digests and spleens following intravenous injection of CD45 Ab (CD45 i.v.) to label circulating white blood cells. The lung-resident (CD45 i.v.–, filled), lung circulating (CD45 i.v.+, open) and splenic (dotted) CD8 T cell compartments were assessed for CD69 expression (top panel). Dot plots of CD69 and CD45 i.v. staining in whole lung CD8 T cells (second panel) and D^b^-NP and PA specific CD8 T cells in each of the lung compartments (bottom two panels). **(C)** Frequencies (left) or numbers (right) of influenza-specific D^b^-NP and D^b^-PA tetramer^+^ cells were quantitated by flow cytometry. Resident memory T cells were defined as (CD8α^+^ CD69^+^ tetramer ^+^ CD45^−^ i.v.–) and circulating (Circ) memory T cells were defined as (CD8α^+^ tetramer^+^ CD45^+^ i.v). **(D)** Numbers of influenza-specific D^b^-NP (top) and D^b^-PA tetramer^+^ cells (bottom) in the spleens were measured by flow cytometry. Central (T_CM_) and effector (T_EM_) memory T cells from the spleen were defined as CD8α^+^ CD44^Hi^ CD127^+^tetramer^+^ and were, respectively, CD62L^Hi/Lo^. **(E–G)** Lung and blood effector T cells were quantified at 10 days post infection. **(E)** Representative FACS-plot of Db-NP or Db-PA tetramer staining in CD8 T cells of the whole lung or blood. **(F)** Frequencies (upper panel) and numbers (lower panel) of influenza-specific D^b^-NP and D^b^-PA tetramer^+^ CD8 T cells in lungs. **(G)** Frequencies of D^b^-NP and D^b^-PA tetramer^+^ CD8 T cells in blood. ^*^p < 0.05 for cKO compared to wt. Data are representative of three experimental replicates, except **(D,G)**.

Peroxisome proliferator-activated receptor gamma (PPAR-γ) is a member of the nuclear hormone receptor superfamily. It is a lipid sensing transcription factor that regulates lipid uptake and glucose metabolism (57). Because of the vital importance of the myeloid compartment to mucosal respiratory infections (54), we investigated a model from mice with a conditional PPAR-γ deletion known to have prolonged recovery from influenza infection in multiple contexts, including obesity (54, 58, 59). In this model, Lyz2-driven Cre expression in PPAR-γ^fl/fl^ animals present with transgene penetrance in macrophages, neutrophils and circulating monocytes; however, only alveolar macrophages expressed significant amounts of PPAR-γ protein. We and others previously found that PPAR-γ in alveolar macrophages suppressed inflammation and accelerated recovery following influenza or RSV infections without affecting viral clearance (54, 59). This genetic model exhibits enhanced acute morbidity and prolonged recovery from influenza infection measured by changes in body weight (Figure 1A).

Given this phenotype, we surmised that the flu infection was more severe in cKO mice and that the local CD8 memory T cell response may reflect this (60). Sixty days following infection, we noticed a selective upregulation of CD69 in the parenchymal CD8 T cell compartment of wt and cKO mice compared to CD8 T cells circulating through the lung or spleen (Figure 1B, top panel). The gating scheme used for resident and circulating flu-specific T cells throughout the manuscript can be seen in Supplementary Figure 1A. We observed an increase in frequency and number of lung-resident CD69^+^ CD8 T cells precluded from circulation from cKO vs. wt animals 60 days post-infection (p.i.) (Figure 1B, second and third panels). Flu-specific polyclonal CD8 T_RM_ cells against D^b^-restricted NP_366−374_ and PA_224−233_ epitopes increased 2-fold each in cKO animals relative to wt controls. This difference was not seen in memory T cells circulating through the lung (Figure 1B, bottom panel and Figure 1C). These changes in local T_RM_ density could not be explained by differences in circulating memory T cells as no changes were observed in the magnitude of T_EM_ or T_CM_ responses in the spleen (Figure 1D). An increase in T_RM_ establishment might readily be explained if cKO animals had a larger effector T cell pool early in the immune response (61). However, in this regard, we found that although cKO and wt littermates had differences in CD8 T_RM_ cell frequency, CD8 effector T cell numbers in whole lungs were unchanged 10 days post-infection (Figures 1E,F). There was a slight increase in frequency of Db-PA CD8 T cells from cKO animals in the peripheral blood (Figures 1E,G), however, as noted, this did not carry over into differences in the number of circulatory T_EM_ or T_CM_ cells. Therefore, Lyz2-driven PPAR-γ deficiency led to enhanced T_RM_ establishment without majorly affecting influenza-specific effector or circulatory memory T cell responses.

### Myeloid PPAR-γ Deficiency Impairs AM Compartment Following Influenza Infection

**Figure 2:**
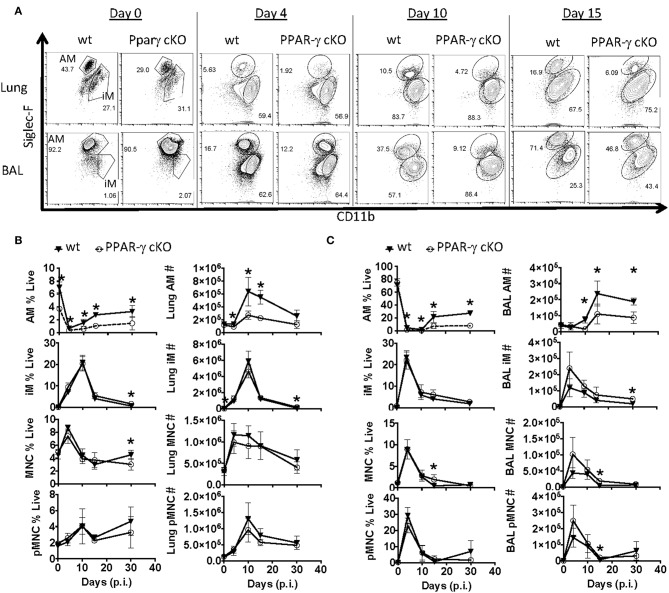
PPAR-γ cKO mice exhibit abnormal alveolar macrophage kinetics following influenza challenge. Wt C57BL/6 and PPAR-γ^f^ (cKO) mice were infected with influenza PR8 virus. Alveolar macrophage (CD64^+^ Siglec-F^Hi^ CD11b^int^), inflammatory macrophage (CD64^+^ Siglec-F^Lo^ CD11b^Hi^), monocyte (CD64^−^ Siglec-F^−^ Ly6G^−^ CD11b^Hi^) and neutrophil (Ly6G^Hi^ CD11b^Hi^) frequencies and numbers were determined by flow cytometry. **(A)** Representative FACS plots of alveolar macrophages (AM) and inflammatory macrophages (iM) within lung (top panel) or BAL fluid (bottom panel). **(B,C)** Frequencies (left) and numbers (right) of alveolar macrophages (AM), inflammatory macrophages (iM), monocytes (MNC), and neutrophils (pMNC) in **(B)** lung and **(C)** BAL at indicated days post-infection (p.i.). ^*^p < 0.05 for cKO compared to wt. Data are compiled from 2 to 3 experimental replicates.

Since the macrophage compartment has been shown to influence T_RM_ number (40, 48), we examined two populations of lung macrophages including fetal-derived alveolar (CD64^+^ CD11b^int^ Siglec F^hi^) and adult monocyte-derived macrophages (CD64^+^ CD11b^hi^ Siglec F^Lo^) prior to and after influenza infection by flow cytometry in whole lung and airway lumen (Figures 2A–C). Gating strategy for myeloid cells in lung compartments is displayed in Supplementary Figure 1B. The pulmonary myeloid compartment showed a kinetic decrease of tissue-resident alveolar macrophage, but not monocyte-derived macrophages or circulating monocyte numbers 4, 10, 15, and even 30 days post-infection in cKO vs. wt lungs and bronchial alveolar lavage fluid (BAL). Together, the data indicated that PPAR-γ in the myeloid compartment is important for AM response kinetics following influenza infection and has moderate effects on the magnitude of other myeloid cell responses in the lung.

### Early AM Depletion Minimally Affects Effector CD8 T Cell Response

**Figure 3:**
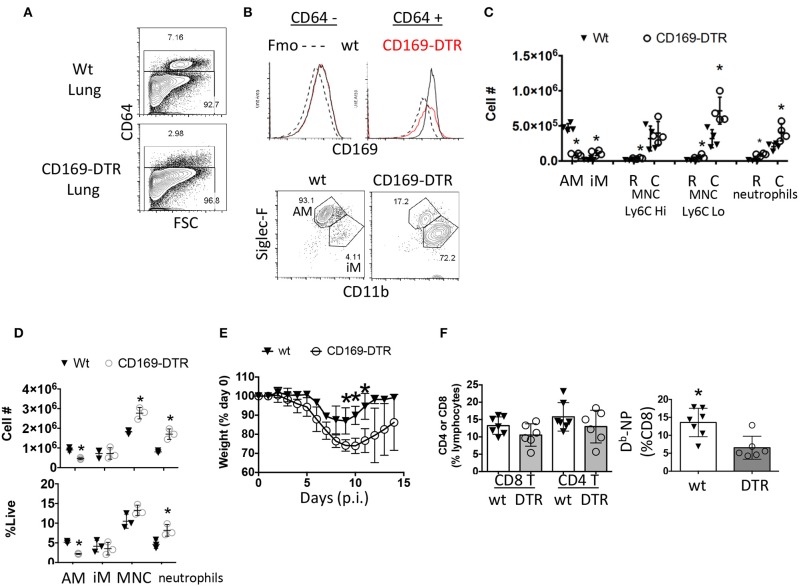
Early ablation of CD169^+^ cells leads to enhanced morbidity without enhancing circulating effector CD8 T cell responses. **(A–C)** Naïve Wt littermates or CD169-DTR mice were treated with DTx then euthanized 4 days later. **(A)** CD64 positive and negative compartments were assessed by flow cytometry. **(B)** CD169 expression in CD64 positive and negative cells from the lung were measured by flow cytometry and compared to a fluorescent-minus-once (Fmo) control for CD169 antibody specificity (top). CD64^+^ cells were classified as AM (Siglec-F^Hi^ CD11b^int^) or iM (Siglec-F^Lo^ CD11b^Hi^) (**B**, bottom). **(C)** Lung resident (R, CD45^−^ i.v.) and circulating (**C**, CD45^+^ i.v.) alveolar macrophages (AM) inflammatory macrophages (iM), monocytes (MNC), and neutrophils were quantitated by flow cytometry. **(D–F)** Wt littermates or CD169-DTR mice were treated with DTx then infected with influenza PR8. **(D)** Lung myeloid cell populations were quantified by flow cytometry at day 3 post-infection. **(E)** Percentage of initial body weight was measured daily for 14 days post-infection (p.i.). **(F)** Percentage of CD4 and CD8 T cells (left) or % of D^b^-NP-specific CD8 T cells from blood PBMCs 10 days post-infection. ^*^p < 0.05 for CD169-DTR compared to wt. Data are representative of 2–3 experimental replicates except **(A–C)**.

Since we have narrowed down the influenza response defect in these mice to alveolar macrophages (59) while observing their frequency changes correlated with T_RM_ establishment, we suspected alveolar macrophages may be playing a heretofore unappreciated role in CD8 T_RM_ differentiation and or maintenance. To assess whether alveolar macrophages were important for T_RM_ establishment, we utilized a transgenic model (CD169-DTR) (51, 52, 62). Parabiosis experiments demonstrate the vast majority of these CD169-expressing cells are tissue-resident macrophages in gut and kidney models (51, 52). With regards to the lung, we reasoned we were primarily depleting resident alveolar macrophages in a well-characterized model (51, 52, 63, 64). To verify this assumption, we monitored ablation efficiency by Immuno-staining myeloid cells from the naïve lungs of CD169-DTR and littermate control animals treated once with Diptheria toxin (DTx). We then examined CD169 expression in CD64 positive macrophages and CD64 negative cells in both the circulation and parenchyma 3 days later (Figure 3A). Higher CD169 expression was evident in the CD64^+^ vs. negative compartment. Importantly, DTx treatment did not cause changes in the low levels of CD169 expression in CD64^−^ cells, suggesting DTx treatment did not majorly affect the CD64^−^ compartment (Figure 3B). The vast majority (93.1%) of CD169^+^ cells in lungs were resident alveolar macrophages that were largely depleted following DTx treatment (Figures 3B,C). Simultaneously, there was an enrichment of monocyte derived macrophages (CD11b^hi^ SiglecF^Lo^ CD64^+^), Ly6C^Lo^ circulating monocytes, and circulating neutrophils (Figure 3C). To understand the model in the context of influenza infection, we administered DTx 1 day prior to infection and characterized lungs 3 days later (Figure 3D). Alveolar macrophages were clearly decreased from the pulmonary compartment in contrast to inflammatory macrophages. As previously reported (63), this was also accompanied by an increase in circulating monocyte and neutrophil numbers as seen in the uninfected animals (Figure 3D). Therefore, within the lung, DTx treatment in CD169-DTR mice rather selectively depleted Siglec F^hi^ alveolar macrophages (CD64^+^ CD11c^hi^ CD11b^int^), while leading to numeric increases in other inflammatory myeloid populations in the presence or absence of infection. This population has been shown to be a CCR2-independent resident population from an embryonic origin that self-maintains through homeostatic proliferation even after depletion by DTx (CD169-DTR mice) or respiratory virus challenge (62).

Macrophage numbers recover by 7 days post DTx treatment in CD169-DTR BALB/c mice leading us to choose every third day to administer DTx (51, 52). As has been previously reported (63), absence of CD169^+^ cells prior to infection led to prolonged recovery of weight loss following influenza infection (Figure 3E). We found a small, but noticeable decrease in Flu-specific D^b^-NP effector cell frequency in the blood in the CD169-DTR model (Figure 3F). Thus, depletion of alveolar macrophages mimicked morbidity in the conditional PPAR-γ knockout animals with impaired macrophage function and did not enhance the magnitude of the CD8 T cell response in the circulation.

### Early AM Depletion Promotes T_RM_ Development

**Figure 4:**
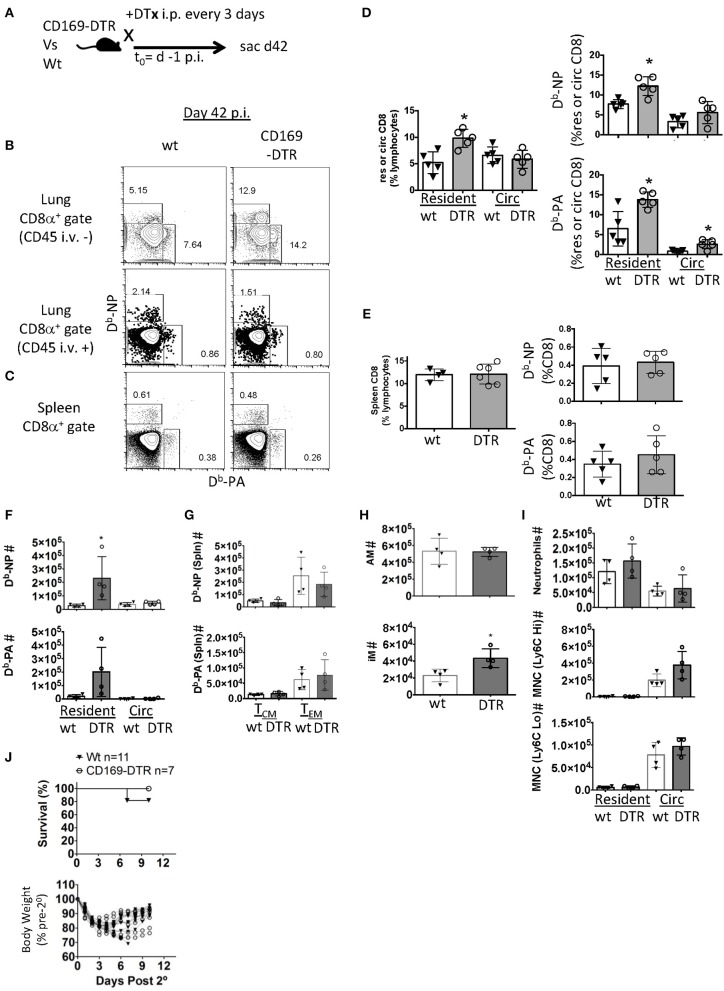
Early alveolar macrophage ablation enhances flu-specific T_RM_ establishment. **(A)** Wt C57BL/6 or CD169-DTR mice were infected with influenza PR8. **(A)** Schematic of experimental design showing that mice were treated with DTx on day minus 1 and every third day, until day 42 p.i. **(B)** Representative FACS plots of lung resident (CD45 i.v.–) or circulating (CD45 i.v.+) D^b^-NP or D^b^-PA memory T cells within CD8 T cell compartment in Wt or CD169-DTR mice. **(C)** Representative FACS plots of splenic D^b^-NP or D^b^-PA memory T cells within the CD8 T cell compartment in Wt or CD169 DTR mice. **(D)** Frequencies of CD8 T cells in lung resident or circulating lymphocytes (left). Frequencies of influenza-specific D^b^-NP and D^b^-PA tetramer^+^ memory T cells in lung resident or circulating (Circ) CD8 T cells (**D**, right). **(E)** Frequency of CD8 T cells in splenic lymphocytes (left). Frequencies of influenza-specific D^b^-NP and D^b^-PA tetramer^+^ memory T cells in splenic CD8 T cells (**E**, right). **(F–J)** C57BL/6 or CD169-DTR mice were infected with influenza PR8. Mice were treated with DTx on day minus 1 and every third day until day 42 p.i. mice were sacrificed or rechallenged with influenza X31 at day 53 pi. **(F)** Numbers of lung resident (CD45 i.v.–) or circulating (CD45 i.v.+) D^b^-NP or D^b^-PA memory T cells at day 53 p.i. **(G)** Numbers of splenic D^b^-NP or D^b^-PA memory T cells at day 53 p.i. **(H)** Alveolar macrophages (AM, top) or inflammatory macrophages (IM, bottom) were quantitated in the lungs. **(I)** Neutrophils, Ly6C^Hi^ or Ly6CLo monocytes in the parenchyma (CD45 i.v.–) or circulation (CD45 i.v.+). **(J)** Mice were treated with FTY720 at day 52 p.i. and re-infected with X-31 on day 53. FTY720 treatment was maintained throughout the challenge phase. Hose survival (top) and weight loss and recovery of individual mice (bottom) were measured to Wt C57. **(B–E)** Antigen-specific lung resident, circulating (Circ) and splenic D^b^-NP and -PA CD8 T cell frequencies were measured. DTx treatment was stopped on day 42 and **(F)** flu-specific lung-circulating, –resident, and **(G)** splenic central and effector memory cells were quantitated on day 53. **(H,I)** Myeloid cells were quantitated in the parenchyma and circulation. **(J)** Mice were treated with FTY720 at day 52 p.i. and re-infected with X-31 on day 53. FTY720 treatment was maintained and survival (top) and weights (bottom) were measured to day 10 following X-31 infection (days post 2°). ^*^p < 0.05 for CD169-DTR compared to wt. Data are representative of three experimental replicates except **(F–J)** which are single experiments.

In contrast to the comparable effector CD8 T cell responses, 6 weeks post-infection, the depletion of alveolar macrophages pre-infection (Figure 4A) resulted in significant increases in flu-specific (NP and PA D^b^-restricted epitopes) CD8 T_RM_ frequencies (Figure 4B). However, AM depletion did not alter memory CD8 T cells against PA and NP epitopes circulating through the lung or in the spleen 42 days after infection with the exception of a minor increase in PA-specific CD8 T cell frequency in the lung vasculature (Figures 4B–E). We wondered if this increased T_RM_ density could enhance protective immunity. To test this, we withdrew DTx treatment 42 days after infection and rechallenged the mice at day 53 to allow the reconstitution of the AM compartment. We observed increased lung CD8 T_RM_, but not circulating CD8 memory T cells or splenic T_CM_ and T_EM_ numbers against both NP and PA epitopes prior to the re-challenge in this model (Figures 4F,G). AM numbers were equivalent between CD169-DTR and littermate controls (Figure 4H). We also observed an increased number of inflammatory macrophages at day 53 with no changes in neutrophils or monocytes either in the parenchyma or circulation (Figure 4I).

To investigate T_RM_ contribution to secondary immunity in this model, PR8-immune mice were treated with S1P1R antagonist (FTY720) to prevent lymphocytes access to the circulation. Mice were then challenged with a high dose of a heterosubtypic influenza A strain (H3N2, X-31). Two of the Wt mice died following high does of X-31 (*n* = 11) while no CD169-DTR mice were lost (*n* = 7), however this was not a statistically significant finding. We also observed no significant differences in changes in weight loss or recovery (Figure 4J). Together the data suggested alveolar macrophage depletion caused enhanced establishment of T_RM_ despite a blunted effector pool, although whether this enhanced T_RM_ presence is associated with increased immune protection requires further studies.

### Late AM Depletion Does Not Enhance T_RM_ Responses

**Figure 5:**
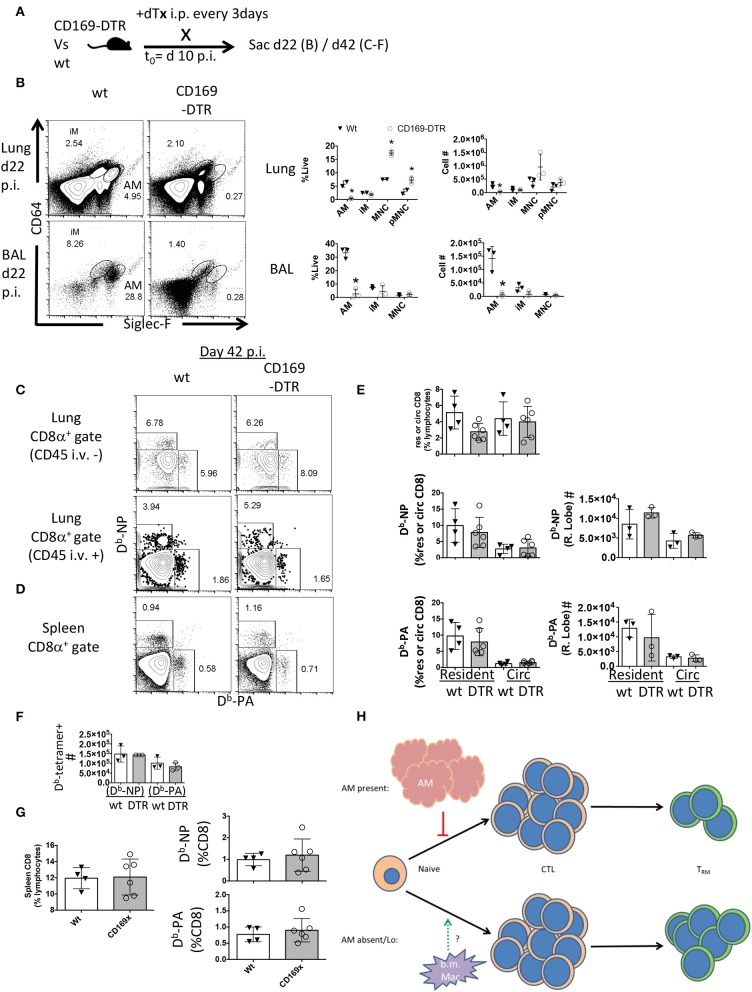
Late CD169^+^ ablation does not affect T_RM_ establishment. Wt C57BL/6 and CD169-DTR mice were infected with influenza. **(A)** Experimental schematic showing DTx regimen began day 10 post-influenza PR8 infection. **(B)** Representative FACS plots of alveolar macrophages (AM) and inflammatory macrophages (iM) in Wt or CD169 DTR mice (left). Frequency (middle) and cell number (right) of AM, iM, and monocytes (MNC) 22 days post influenza infection in the Lung (top) and BAL fluid (bottom). **(C)** Representative FACS plots of influenza-specific D^b^-NP and D^b^-PA tetramer^+^ memory resident (CD45 i.v.–) and circulating (CD45 i.v.+) CD8 T cells in the lung 42 days post-infection. **(D)** Representative FACS plots of D^b^-NP and D^b^-PA tetramer^+^ memory. **(E)** Frequencies of lung CD8 T cells in lung lymphocyte populations in the parenchyma (resident) or circulation (Circ) (top) or of D^b^-tetramer positive memory T cells in the CD8 compartments (left) with Numbers of same (right). **(F)** Numbers of splenic D^b^-NP or D^b^-PA memory CD8 T cells at day 42 p.i. **(G)** Frequencies of CD8 T cells from lymphocytes in the spleen (left) or D^b^-NP or D^b^-PA memory T cells in the CD8 compartment at day 42 p.i. (right). **(H)** Model indicating the early, but not late presence of resident alveolar macrophages (AM) regulates the CD8 resident memory T cell compartment size without affecting magnitude of effector (CTL) response. Data are representative of three experimental replicates, except **(F)** which is a single experiment. ^*^p < 0.05 for CD169-DTR compared to wt.

The preceding experiments did not indicate when alveolar macrophages might be playing a role to limit T_RM_ establishment or maintenance. To address whether alveolar macrophages are needed early vs. late in the effector response to influence local T cell immunity, we next depleted alveolar macrophages 10 days post-infection and maintained the DTx regimen until d42 post-infection (Figure 5A). Following 12 days of this regimen, AM were depleted 100-fold in the BAL and 20-fold in the lung (Figure 5B). This was associated with a skewing of circulating monocyte frequencies, but not numbers (Figure 5B). In contrast to the effects of early alveolar macrophage depletion, loss of CD169^+^ cells during T cell contraction did not significantly change the establishment of Flu-specific T_RM_, lung-circulating, or splenic memory CD8 T cells (Figures 5C–G). Since these results, with respect to local resident memory CD8 T cells, conflict with models ablating CCR2-dependent bone marrow-derived mucosal macrophages (40), it is therefore plausible that embryonic-derived resident macrophages and inflammatory macrophages have distinct functions in the differentiation and or maintenance of CD8 T_RM_ (Figure 5H).

## Discussion

Our findings demonstrate T_RM_ differentiation is regulated early by tissue-resident alveolar macrophages largely independent of the magnitudes of either the effector CTL response or circulating memory T cell pools. This study therefore suggests alveolar macrophages regulate an intercellular immune-network unique to the inflamed tissue and, under certain circumstances, might influence local secondary immunity by governing mucosal T_RM_ density. Conversely, previous studies have revealed that CCR2-dependent monocytes and macrophages are important in the development of CD8 T_RM_ following infection (40, 47). Thus, it is possible that bone-marrow and embryonic-derived macrophages exhibit opposing roles for T_RM_ differentiation (40, 62). Alternatively, macrophage subsets could differentially influence local adaptive immune responses dependent on location or nature of the pathogen-specific response. Though we did not narrow observations to macrophage-intrinsic vs. extrinsic in this study, mechanistically, we find a role for PPAR-γ in the myeloid compartment in limiting T_RM_ density, potentially contributing to blunted secondary and or heterosubtypic immunity. We have independently found that PPAR-γ from alveolar macrophages serves to limit immune response-induced long-term damage following influenza infection demarking this pathway in macrophages as a signaling axis for immunity and pathology (unpublished).

Though we did not find a role for enhanced immunity in mice with early macrophage depletion under the tested conditions, this model likely abolishes any innate memory features that AM develop from viral infections in an effector CD8 T cell-help dependent manner (65, 66). Trained immunity of local innate cells contribute to subsequent responses long after a primary infection. Since we only tested one viral dose in the secondary challenge model, more investigation is required to determine whether protective immunity is enhanced when AM are absent early from a primary response and whether this is dependent on changes in T_RM_ density.

CD8 T cell Immune responses to influenza infection primarily involve the lung and lung-draining mediastinal lymph nodes (67). Peripheral macrophages have not been reported to be involved in CD8 T cell responses to influenza (63). Given that macrophages are not playing a role in peripheral memory T cell differentiation, in contrast to the local infection site in this study, we think it likely that increases in CD8 T_RM_ in our model are from depleting embryonic-derived alveolar macrophages (62). There is also a transient influx of neutrophils into lungs of diphtheria toxin treated CD169-DTR animals compared to wt while not affecting DCs in lung draining lymph nodes (63, 64). Notably, acute neutrophil depletion, at a time when we observed AM playing a role, has shown no relevance to T_RM_ differentiation or maintenance in influenza models despite delaying CTL recruitment (68, 69). Further, macrophages in secondary lymphoid tissue aid in transferring antigen to cross-priming DCs (70). Therefore, we would expect opposite results with regards to long-term T_RM_ establishment if non-local macrophages were playing a major role in that regard (19). We find it more plausible that the loss of embryonic-derived alveolar macrophages released homeostatic controls gearing the lung environment to further favor T_RM_ differentiation.

We do not anticipate that effector T cells are interacting directly with AM given that retained T_RM_ are eventually found in the interstitial tissue and alveolar macrophages are confined within the smallest of airways. Yet, we do not discount this possibility for memory T cells found within alveoli (44, 60, 71, 72). Rather, together with others', our data indicates an abrupt loss of resident macrophage function or number results in this niche temporarily being replaced by monocyte-derived macrophages that migrate into tissues and positively influence T_RM_ differentiation and or maintenance (40, 48). More thorough studies addressing the leukocyte kinetics, direct links, and model disparities are needed. Our data do not refute a model whereby embryonic-derived alveolar macrophages limit known niches for T_RM_ by limiting the damage or disrepair of local inflamed tissue following respiratory viral clearance (60, 73–75).

Surprisingly, late ablation of alveolar macrophages (DTx treatment at day 10), did not influence T_RM_ differentiation nor was their continual depletion necessary when ablation was initiated prior to infection (Figure 4F). Collectively, this suggests alveolar macrophages existing prior to pathogen encounter play an early role in imposing their limits on T_RM_ programming. The direct or indirect mechanisms by which early T_RM_ differentiation is regulated by alveolar macrophages is currently being pursued. Ultimately, altering T_RM_ density through manipulation of the local macrophage pool may circumvent patient-specific roadblocks, such as an individual's HLA alleles or T cell repertoires, which restrict off-the-shelf immunotherapies.

## Data Availability Statement

All datasets generated for this study are included in the manuscript/Supplementary Files.

## Ethics Statement

The studies involving animals were fully reviewed and approved by the internal animal care and use committee (IACUC, approval #A00002035) guidelines at the Mayo Clinic (Rochester, MN).

## Author Contributions

Experiments were designed by NG, SH, and JS. NG and SH performed and analyzed the experiments. NG and JS prepared the manuscript. All the other authors provided substantial insight that drove experimental design.

### Conflict of Interest

The authors declare that the research was conducted in the absence of any commercial or financial relationships that could be construed as a potential conflict of interest.

## Supplementary Material

Supplementary Figure 1 Gating scheme for circulating and parenchymal myeloid cells and T_RM_. 53 days post-infection per DTx regimen in Figures 4F–I, lung digests (Wt shown) were performed after intravenous labeling of circulating leukocytes with anti CD45. **(A)** Following lymphocyte gating in lung samples and exclusion of doublets, viable (ZomB dye Lo) CD8 T cells were gated and compartmentalized into CD69^+^CD45 i.v.– (resident) or CD45 i.v.+ (circulating) populations (left) and D^b^-NP and D^b^-PA tetramer positive memory CD8 T cells in each compartment (right). **(B)** Myeloid cells from the same experiment were examined after excluding doublets from total lung cells and gating on live cells (ZomB dye Lo). Alveolar (AM) and inflammatory (iM) macrophages were segmented by Siglec-F and CD11b expression in cells expressing CD64 (top panel). Ly6G^Hi^ cells were examined for CD11b expression and classified as neutrophils (pMNC) and separated into resident vs. circulating on basis of CD45 i.v. staining (–resident, + circulating; middle right panel). Monocytes (MNC) were CD11b^Hi^ CD64^−^ cells excluded for DCs (CD11c^Hi^ MHCII^Hi^), neutrophils (Ly6G^Hi^+ CD11b^Hi^), and eosinophils (CD64^−^ Siglec-F^−^). Monocytes were compartmentalized by Ly6C expression and whether they had access to the circulation (CD45 i.v.+) or not (CD45 i.v.–).
