# Supplementary figures and images for: Tissue-Resident Macrophages Limit Pulmonary CD8 Resident Memory T Cell Establishment
Source: Front Immunol. 2019 Oct 10;10:2332. doi: 10.3389/fimmu.2019.02332 (PMC6797929; doi:10.3389/fimmu.2019.02332)

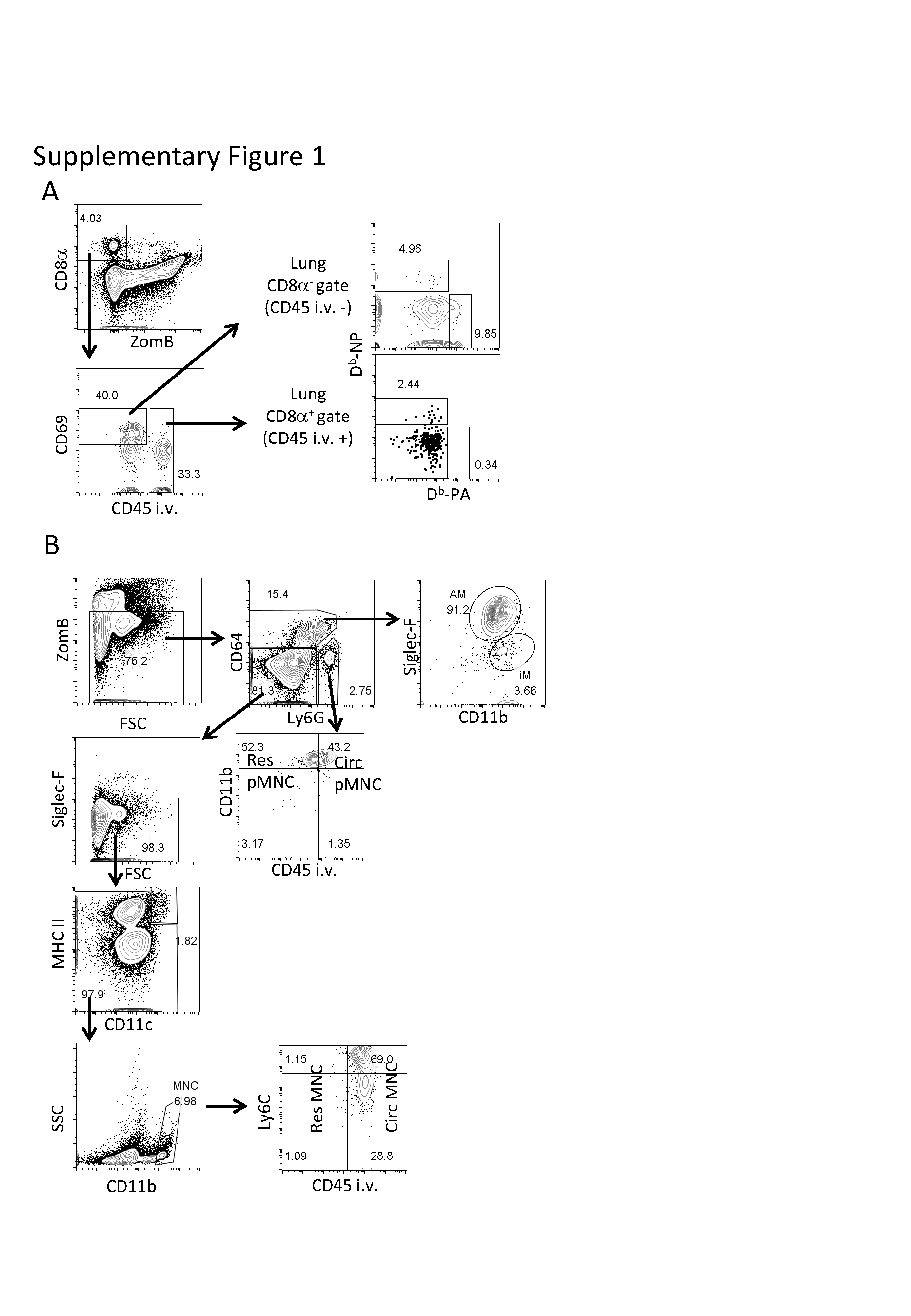

Supplement: Supplementary Figure 1 — Gating scheme for circulating and parenchymal myeloid cells and TRM. 53 days post-infection per DTx regimen in Figures 4F–I, lung digests (Wt shown) were performed after intravenous labeling of circulating leukocytes with anti CD45. (A) Following lymphocyte gating in lung samples and exclusion of doublets, viable (ZomB dye Lo) CD8 T cells were gated and compartmentalized into CD69+CD45 i.v.– (resident) or CD45 i.v.+ (circulating) populations (left) and Db-NP and Db-PA tetramer positive memory CD8 T cells in each compartment (right). (B) Myeloid cells from the same experiment were examined after excluding doublets from total lung cells and gating on live cells (ZomB dye Lo). Alveolar (AM) and inflammatory (iM) macrophages were segmented by Siglec-F and CD11b expression in cells expressing CD64 (top panel). Ly6GHi cells were examined for CD11b expression and classified as neutrophils (pMNC) and separated into resident vs. circulating on basis of CD45 i.v. staining (–resident, + circulating; middle right panel). Monocytes (MNC) were CD11bHi CD64− cells excluded for DCs (CD11cHi MHCIIHi), neutrophils (Ly6GHi+ CD11bHi), and eosinophils (CD64− Siglec-F−). Monocytes were compartmentalized by Ly6C expression and whether they had access to the circulation (CD45 i.v.+) or not (CD45 i.v.–). [file Image_1.TIFF]
